# Telomere shortening impairs alveolar regeneration

**DOI:** 10.1111/cpr.13211

**Published:** 2022-03-11

**Authors:** Xin Zhang, Mengting Shi, Xi Zhao, Ennan Bin, Yucheng Hu, Nan Tang, Huaping Dai, Chen Wang

**Affiliations:** ^1^ Department of Pulmonary and Critical Care Medicine China‐Japan Friendship Hospital Affiliated to Capital Medical University Beijing China; ^2^ Department of Pulmonary and Critical Care Medicine National Clinical Research Center for Respiratory Diseases Beijing China; ^3^ Institute of Respiratory Medicine Chinese Academy of Medical Sciences Beijing China; ^4^ National Institute of Biological Sciences Beijing China; ^5^ Beijing Advanced Innovation Center for Imaging Theory and Technology & Academy for Multidisciplinary Studies Capital Normal University Beijing China

## Abstract

**Objectives:**

Short telomeres in alveolar type 2 (AT2) cells have been associated with many lung diseases. The study aimed to investigate the regeneration capacity of AT2 cells with short telomeres by knocking out *Tert* in mice (G4 *Tert* ^*−/−*^) from the whole to the cellular level.

**Materials and Methods:**

The lung injury model of mice was established by left pneumonectomy (PNX). The proliferation and differentiation of AT2 cells were observed by immunofluorescence staining in vivo and in vitro. The difference of the gene expression between control and G4 *Tert* ^−/−^ group during the regeneration of AT2 cells was compared by RNA sequencing. The expression of tubulin polymerization promoting protein 3 (TPPP3) was reduced by adeno‐associated virus delivery.

**Results:**

The alveolar regeneration in G4 *Tert* ^*−/−*^ mice was impaired after PNX‐induced lung injury. The regulation of cytoskeleton remodelling was defective in G4 *Tert* ^*−/−*^ AT2 cells. The expression of TPPP3 was gradually increased during AT2 cell differentiation. The expression level of TPPP3 was reduced in G4 *Tert* ^*−/−*^ AT2 cells. Reducing TPPP3 expression in AT2 cells limits the microtubule remodelling and differentiation of AT2 cells.

**Conclusion:**

Short telomeres in AT2 cells result in the reduced expression level of TPPP3, leading to impaired regeneration capacity of AT2 cells.

## Graphical abstract

Short telomeres in alveolar type 2 (AT2) cells have been associated with many lung diseases. The study aimed to investigate the regeneration capacity of AT2 cells with short telomeres by knocking out *Tert* in mice (G4 *Tert* ^*−/−*^) from the whole to the cellular level. Our findings suggest that short telomeres in AT2 cells result in the reduced expression level of TPPP3, leading to impaired regeneration capacity of AT2 cells.

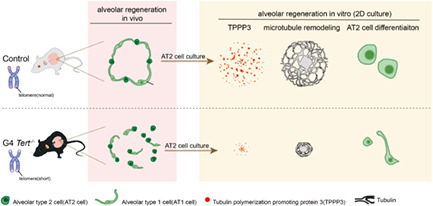

## 1 INTRODUCTION

Telomeres are composed of TTAGGG repeated DNA sequences and shelterin proteins, which cover the ends of chromosomes to protect them from degradation and DNA damage reactions.^1^, ^2^ Telomeres progressively shorten with age in humans and mice, eventually triggering senescence and apoptosis. The length of telomeres is maintained by telomerase, a specialized reverse transcriptase with two components: the RNA template telomerase RNA component (TERC) and the catalytic component telomerase reverse transcriptase (TERT).^3^ Mutations in *TERT* and/or *TERC* can lead to the deficient telomerase, and ultimately to telomere shortening.

Short telomeres have been linked to lung diseases for decades,^4^, ^5^, ^6^ and are strongly associated with abnormal alveolar type 2 (AT2) cells. In patients with idiopathic pulmonary fibrosis (IPF), the telomere lengths of AT2 cells were shorter than those of surrounding cells, and the telomere lengths of AT2 cells in fibrotic areas were even shorter than in non‐fibrotic areas.^7^ Moreover, the telomere lengths of AT2 cells in patients with *TERT* mutations were significantly shorter than in normal patients. In mice, short telomeres enhanced susceptibility to pulmonary fibrosis and emphysema after exposure to bleomycin or cigarettes.^8^, ^9^ Overexpression of *Tert* in AT2 cells increased telomere length and the proliferation of AT2 cells, also significantly reducing DNA damage, apoptosis and senescence.^10^ These studies demonstrated that short telomeres resulted in abnormal AT2 cells, eventually driving the development of lung diseases.

There are two types of alveolar epithelial cells: large and flat alveolar type 1 (AT1) cells, which are responsible for gas exchange, and AT2 cells that function as alveolar stem cells.^11^, ^12^ AT2 cells are known to proliferate and differentiate into AT1 cells after lung injury to maintain the normal alveolar structure and function.^13^, ^14^ It has been reported that impaired regeneration of AT2 cells can lead to the lung diseases.^15^, ^16^, ^17^

The cytoskeleton is a complex, dynamic network of protein filaments in the cytoplasm of all cells. The cytoskeleton has been shown to be involved in construction of cells morphology, maintenance of structural integrity, cell division and migration.^18^, ^19^, ^20^ Eukaryotes comprise three cytoskeletal polymers, microtubules, actin and intermediate filaments. It has been reported that cytoskeleton remodelling is an indispensable part in the cell growth and maturation.^19^, ^21^ Microtubules participate in the cell proliferation, the mitotic spindle formation and cellular organization by locating organelles and establishing the polarity of various cells in plants and animals. Microtubules also provide tracks for motor proteins that catalyse the movement of organelles, transport vesicles and other structures.^22^, ^23^, ^24^ The tubulin polymerization promoting protein 3 (TPPP3) is one of the members of tubulin polymerization promoting protein family members (TPPPs), which are regulators of microtubule dynamic that has microtubule bundling activity.^25^, ^26^, ^27^ Previous studies showed that TPPPs are required for central and peripheral nerve regeneration.^28^, ^29^ However, the relationship between cytoskeleton and alveolar regeneration has not been studied yet.

Nowadays, the vital role of alveolar regeneration in the lung diseases is gradually recognized, but few studies on the short telomere‐related alveolar regeneration have been fully elaborated, and the researches on mechanism of impaired regeneration of AT2 cells of short telomere are scarce. Therefore, it is necessary to investigate the effect of short telomeres on alveolar regeneration, and further explore the mechanism to provide new ideas for the diagnosis and treatment of lung diseases. Here, we established a murine model of short telomeres with *Tert* knockout mice after four generations of inbreeding (G4 *Tert* ^*−/−*^) and investigated the regeneration of AT2 cells in G4 *Tert* ^*−/−*^ mice after pneumonectomy (PNX). We demonstrated that the telomere length in G4 *Tert* ^*−/−*^ AT2 cells was shortened. The proliferation and differentiation of AT2 cells were both impaired in G4 *Tert* ^*−/−*^ AT2 cells. We found that the expression level of TPPP3, which regulates microtubule remodelling during AT2 cell differentiation, was decreased in G4 *Tert* ^*−/−*^ AT2 cells. The reduced TPPP3 expression results in impaired differentiation of G4 *Tert* ^*−/−*^ AT2 cells.

## 2 MATERIALS AND METHODS

### 2.1 Mice

*Sftpc*‐CreERT2 (*Sftpc*‐CreER); *Rosa26*‐CAG‐mTmG (*Rosa26*‐mTmG), *mTert* ^−/−^ and pdgfα^+^‐eGFP have been described previously.^30^, ^31^ In brief, homozygote *Tert* ^−/−^ mice were crossed with *Sftpc*‐CreER; *Rosa26*‐mTmG mice to produce *Sftpc*‐CreER; *mTert* ^−/+^; *Rosa26*‐mTmG, and these mice were crossed to generate the first generation of *Sftpc*‐CreER; *mTert* ^−/−^; *Rosa26*‐mTmG (G1 *Tert* ^−/−^). The fourth generation of *Sftpc*‐CreER; *mTert* ^−/−^; *Rosa26*‐mTmG (G4 *Tert* ^−/−^) mice were inbred from G1 *Tert* ^−/−^. The mice were provided food and water ad libitum and held at 24–26°C and humidity of 50%–60%. All mice experiments were performed in accordance with the guidelines for the use and care of laboratory animals of National Institute of Biological Sciences (NIBS). Male transgenic *Sftpc*‐CreER; *Rosa26*‐mTmG (control), *Sftpc*‐CreER; *mTert* ^−/−^; *Rosa26*‐mTmG (G4 *Tert* ^−/−^) and pdgfα^+^‐eGFP (Pdgfα^+^ stromal cells) aged 8–10 weeks were studied. The mice were administered four doses of tamoxifen (TAM, Sigma T5648‐1G) intratracheally before PNX and the isolation of AT2 cells.

### 2.2 Pneumonectomy

As previously described,^32^ the control and G4 *Tert* ^*−/−*^ mice were anaesthetized by intraperitoneal injection of 0.8% pentobarbital sodium (0.1 ml/10 g body weight). An incision was made at the fourth intercostal ribs, and the left lung was removed. The mice were sacrificed on post‐PNX Days 0, 5, 14 and 21 days to assess changes in lung morphology and gene expression of RNA sequencing (RNA‐seq).

### 2.3 Haematoxylin and eosin staining

The lungs in control and G4 *Tert* ^*−/−*^ mice were fixed with 4% paraformaldehyde (PFA) for 24 h, then paraffin‐embedded and sectioned. The experiment followed the standard Haematoxylin and eosin (H&E) protocol. In brief, after dewaxing and rehydrating, the slides were stained with haematoxylin (Abcam, ab150678) for 2 min, and eosin (Sigma, ht110280) for 1 min. The slices were dehydrated and mounted with natural resin.

### 2.4 Immunofluorescence staining

The lung tissues and cells of mice were fixed with 4% PFA under 4°C for 24 h, and OCT embedding was done after 30% sucrose cryoprotection. Then immunofluorescence staining was performed for frozen sections of 20‐μm thickness. Briefly, the sections were antigen‐repaired with citric acid and blocked at room temperature for 1 h. Then the sections were incubated with primary antibody at 4°C overnight in darkness. The primary antibodies used are as follows: chicken‐anti‐GFP (1:500, ab13970‐100), rabbit‐anti‐HOPX (1:100, sc‐30216), mouse‐anti‐HOPX (1:100, sc‐398703), mouse‐acetylated tubulin (1:100, T6793, Sigma), rat‐anti‐Ki67 (1:100, ab15580), rabbit‐anti‐sftpc (1:200, ab3786), rabbit‐anti‐TPPP3 (1:50, NBP2‐13469) and phalloidin (1:50, ab176757). The secondary antibody was diluted at 1:500 and incubated for 3 h at room temperature. The nucleus was stained with DAPI. The sections were mounted with glycerin.

### 2.5 Isolating AT2 cells and pdgfα^+^ stromal cells

For cell culture and fluorescence in situ hybridization (FISH), control, G4 *Tert* ^−/−^ and pdgfα mice were sacrificed in homeostasis. For RNA‐seq, control and G4 *Tert* ^−/−^ mice were sacrificed on post‐PNX Days 0 and 14. Then the digestive enzyme solution containing neutral protease (5 U/ml, LS02111, Worthington) and DNase I (0.33 U/ml, 10104159001, Roche) were injected into the trachea. After incubating for 45 min in digestive enzymes solution, the lung was cut into small pieces and vortexed on low power for 10 min. The cell mixture was filtered through 100 and 40‐μm strainers and incubated in red blood cell lysis buffer for 5 min. The cells were stained with antibodies as follows: PE‐Cy™7 rat anti‐mouse CD31 (1:400, B&D, 561410) and PE‐Cy™7 rat anti‐mouse CD45 (1:400, B&D, 552848). AT2 cells and pdgfα^+^ stromal cells were sorted for selecting GFP^+^CD31^−^CD45^−^ cells using the single‐cell module on the BD fluorescence‐activated cell sorting (FACS) Aria fusion I appliance.

### 2.6 Fluorescence in situ hybridization

The suspension of AT2 cells in control and G4 *Tert* ^*−/−*^ mice sorted by FACS were fixed with PFA, and dropped on slides at a concentration of 1000 cells/50 μl, then baked at 55°C overnight. Slides were washed in PBS for 15 min and then incubated in RNase A solution (Sigma) for 1 h. After washing in 2× SSC (Thermo) for 30 min, the slides were immersed in 0.005% pepsin (Sigma) for 4 min at 37°C. Then gradient alcohol dehydration was performed as followed: 1 min each in 70%, 85% and 100% alcohol. The slides were incubated in telomere PNA probe solution (Panagene), denatured at 85°C for 10 min, and then kept at room temperature for 1 h in darkness. The slides were washed in washing solution of pH 7.4 containing 20 mM Na_2_HPO_4_ (Sigma), 20 mM Tris, 60% formamide (Sigma), 0.1% μg/ml salmon sperm DNA (Sigma) and 2× SSC for 10 min. The nucleus was stained with DAPI. The sections were mounted with glycerin.

### 2.7 Cell culture

For alveolar organoid culture, the control and G4 *Tert* ^−/−^ AT2 cells were sorted by FACS as described above, and then separately cultured with normal pdgfα^+^ stromal cells in transwell plates. The cell mixture in a well consisted of 5000–10,000 AT2 cells and 250,000–300,000 pdgfα^+^ stromal cells in total 90 μl cell suspension. The matrigel (1:100, Corning, 356231) and rock inhibitor (1:1000, Selleckchem, S8448) were added to improve cell growth. The 600 μl cell culture medium was added to the well. Then cells were cultured at 37°C with 5% CO_2_ concentration, and the medium was changed every other day.

For AT2 cell culture on glass (2D culture system), the matrigel was added into 24‐well plates at 300 μl/well before sorting AT2 cells by FACS. Sterilized round glasses were placed into wells and incubated overnight at 37°C. AT2 cells were resuspended with medium at the concentration of 1 × 10^6^/ml. After the matrigel was discarded, cell suspension of 400 μl was added to each well. The cells were collected at Days 1, 3, 5 and 7 for immunofluorescence staining.

### 2.8 RNA sequencing analysis

RNA‐seq was performed in the sequencing centre at NIBS. The mRNA enrichment and ribosomal RNA removal were performed, then the cDNA was synthesized and the adapter‐linked sequencing library was prepared. The library was then sequenced on a high‐throughput platform (Illumina, NextSeq 2000, USA) to a reading depth of 20 million reads per sample, and 86 bp single‐end reads were generated. After removal of adaptors and low‐quality reads, clean reads of each sample were mapped to the mouse reference genome (UCSC mm10) via STAR software (version 2.7.2a) with default parameters, then all the resulting Binary Alignment MapBAM files were used for gene expression level quantification via feature Counts software (version 1.6.4) based on the annotated gene structures (UCSC mm10) with default parameter settings. After that, gene expression profiles of all samples were subjected to perform differential gene expression analysis, and differentially expressed genes (DEGs) between any two conditions were identified via edgeR package (version 3.28.1) based on the overdispersed Poisson model and empirical bays methods. Next, DEGs with fold change ≥1.5 and adjusted *p*‐value <0.05 were screened for further analysis.

### 2.9 Adeno‐associated virus delivery

After the endotracheal cannula was inserted into the trachea of the anaesthetized mice, the AAV2/9 virus expressing *Tppp3* shRNA (GCGAAATCTGCTAGAGTAA) or scramble shRNA (TTCTCCGAACGTGTCACGT) was delivered into the mouse lungs (Obio Technology company China, Shanghai). Each mouse was treated with 1 × 10^11^ copies of the viral genome diluted in 50 μl of warm sterile saline. Fourteen days after the delivery of the AAV2/9 virus, AT2 cells of these mice were collected for quantitative RT‐PCR analysis and 2D cell culture.

### 2.10 Statistical analysis

All values are expressed as mean ± SEM. The Student's *t*‐test of variance was used to analyse the differences between groups. Statistical analysis was performed using GraphPad Prism software. *p* < 0.05 was considered statistically significant.

## 3 RESULTS

### 3.1 The telomere length is reduced in AT2 cells of *Tert* knockout mice

**FIGURE 1:**
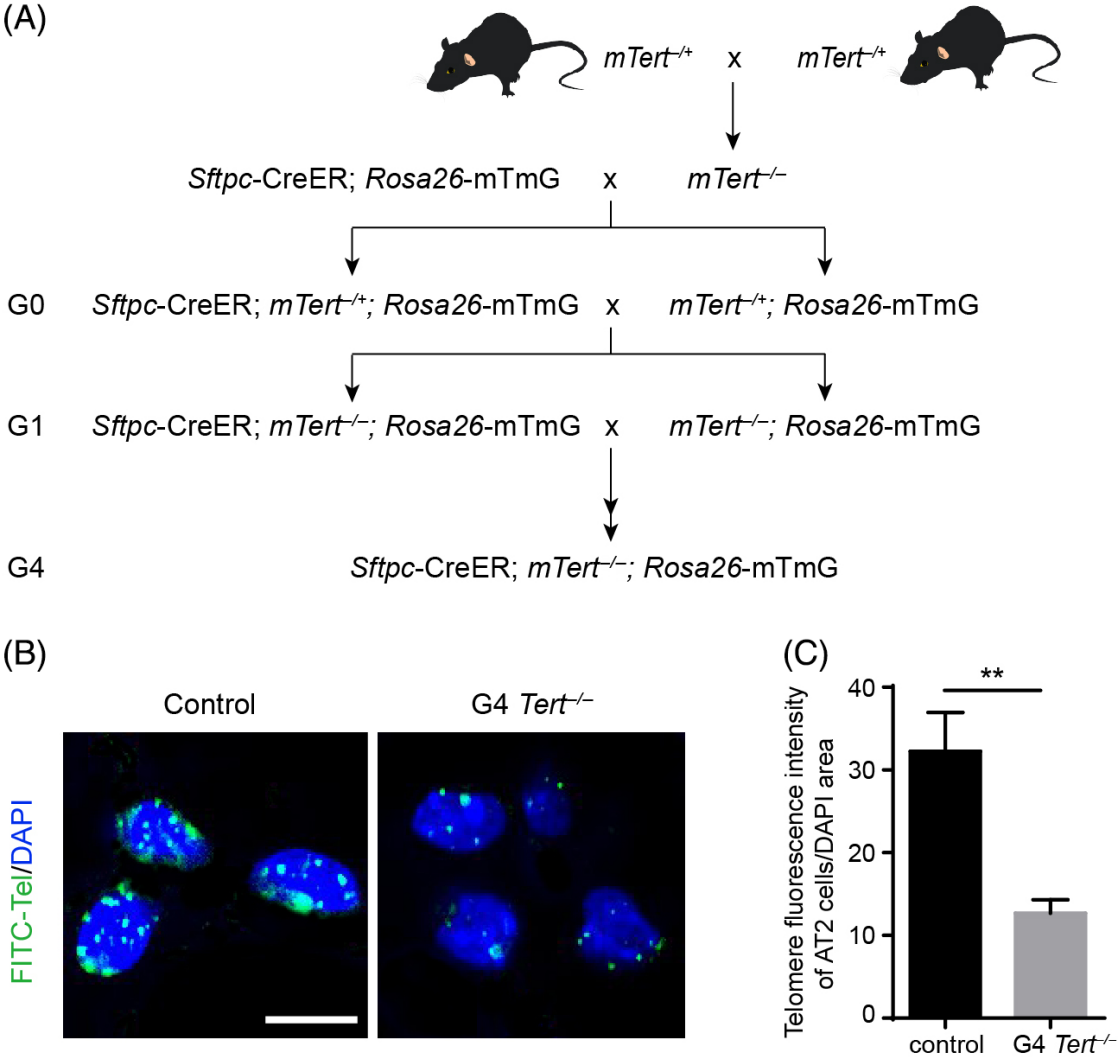
The telomere length of G4 *Tert* ^*−/−*^ AT2 cells is shortened. (A) The schematic diagram of breeding strategy used to generate G4 *Tert* ^*−/−*^ mice. (B) Image of fluorescence in situ hybridization of telomeres of control and G4 *Tert* ^*−/−*^ AT2 cells. (C) Quantification of the telomere fluorescence intensity of control and G4 *Tert* ^*−/−*^ AT2 cells (mean ± SEM, *n* = 10). Student's *t*‐test. *p < 0.05, **p < 0.01, ***p < 0.001, n.s., not significant. Scale bars are 5 μm (B)

We generated *Tert* ^*−/−*^ null mice by genetically deleting the telomerase reverse transcriptase. To specifically lineage trace the cellular behaviours of AT2 cells in the lung, we generated a *Sftpc*‐CreER; *mTert* ^*−/−*^; *Rosa26*‐mTmG mouse model (Figure 1A). In this model, AT2 cells can be lineage labelled by green fluorescent protein (GFP) after intraperitoneal administration of tamoxifen (TMX). In addition, these mice were inbred to the fourth generation of *Sftpc*‐CreER; *mTert* ^*−/−*^; *Rosa26*‐mTmG (G4 *Tert* ^*−/−*^) based on previous studies,^33^ while *Sftpc*‐CreER; *Rosa26*‐mTmG mice were used as control mice (control).

To determine the lengths of telomeres in AT2 cells of G4 *Tert* ^*−/−*^ mice, we isolated AT2 cells from control and G4 *Tert* ^*−/−*^ mice and performed quantitative fluorescence in situ hybridization. As shown in Figure 1B, the nuclei of AT2 cells in the control mice were labelled with a large number of green telomeric DNA probes. The intensity of fluorescence was significantly reduced in G4 *Tert* ^*−/−*^ AT2 cells (Figure 1C). Therefore, we concluded that the length of telomeres in G4 *Tert* ^*−/−*^ AT2 cells was significantly reduced.

### 3.2 The regeneration of G4 *Tert* ^*−/−*^ AT2 cells is impaired

**FIGURE 2:**
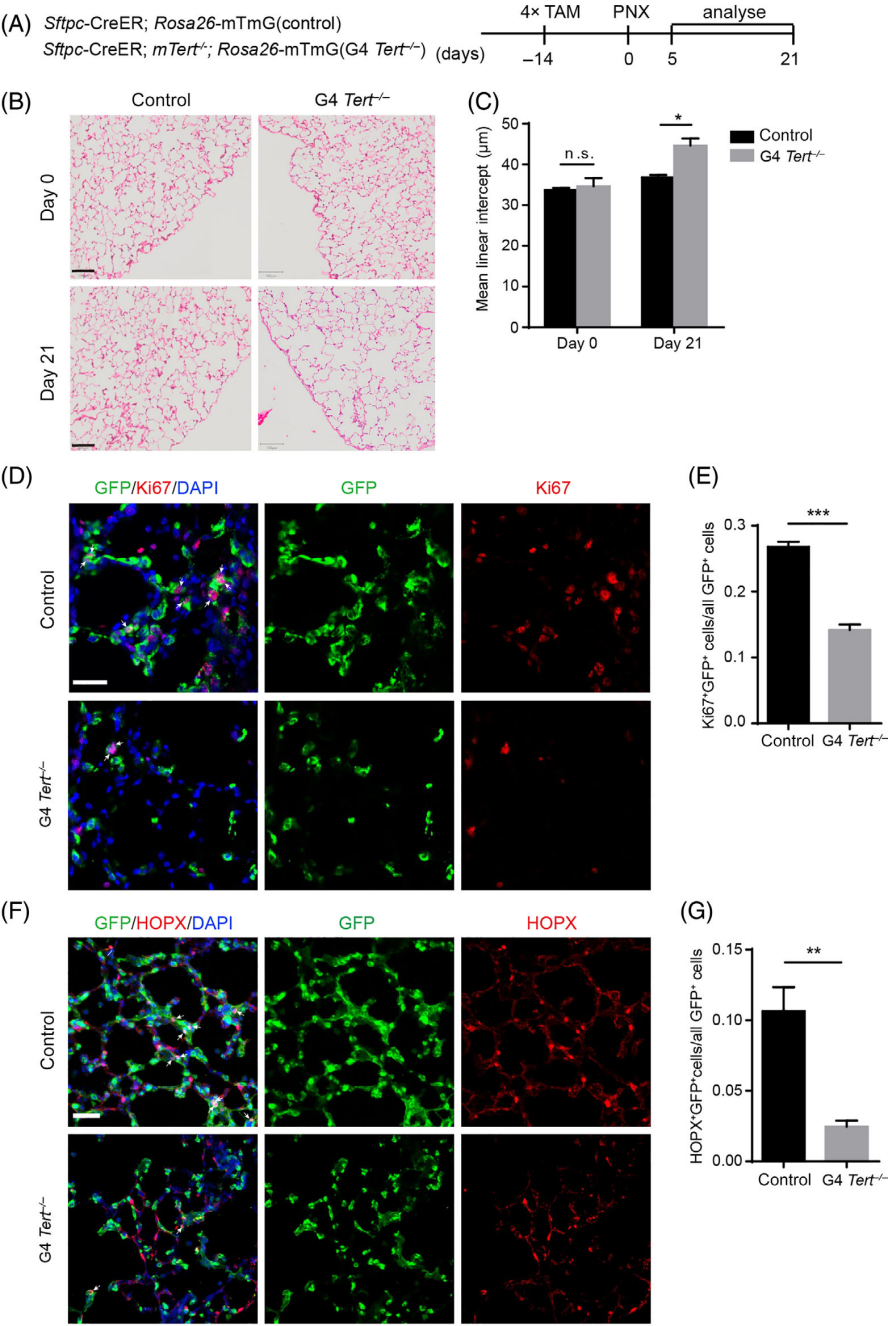
The alveolar regeneration in G4 *Tert* ^*−/−*^ mice is impaired. (A) Analysis of control and G4 *Tert* ^*−/−*^ mice was performed on post‐PNX Days 0, 5 and 21. (B) Representative H&E photomicrographs of lung sections from control and G4 *Tert* ^*−/−*^ mice on post‐PNX Days 0 and 21. (C) Quantification of the mean linear intercept (MLI) (μm) of control and G4 *Tert* ^*−/−*^ mice on post‐PNX Days 0 and 21 (mean ± SEM, *n* = 3). (D) Lungs were stained with antibodies against Ki67 and GFP on post‐PNX Day 5. White arrowheads indicate proliferating AT2 cells. (E) Quantification of the percentage of Ki67^+^GFP^+^ cells/all GFP^+^ cells (mean ± SEM, *n* = 3). (F) Lungs were stained with antibodies against HOPX, GFP on post‐PNX Day 21. White arrowheads indicate the AT1 cells derived from lineage labelled AT2 cells. (G) Quantification of the percentage of HOPX^+^GFP^+^ cells/all GFP^+^ cells (mean ± SEM, *n* = 3). Student's *t*‐test. *p < 0.05, **p < 0.01, ***p < 0.001, n.s., not significant. Scale bars represent 50 μm (D,F), 100 μm (B)

We then analysed the lung morphology by H&E staining of control and G4 *Tert* ^*−/−*^ mice in homeostasis and after a left PNX (Figure 2A). PNX can induce alveolar regeneration in mice. The H&E staining of the lung shows no observed differences between non‐injured control and G4 *Tert* ^*−/−*^ mice (Figure 2B). We also observed the lung morphology of control and G4 *Tert* ^*−/−*^ mice in lung injury model. On post‐PNX Day 21, H&E staining revealed enlarged alveoli of G4 *Tert* ^*−/−*^ mice compared to the alveoli of control mice (Figure 2B,C).

To further investigate the regeneration capacity of AT2 cells in G4 *Tert* ^*−/−*^ mice after PNX, the proliferation of AT2 cells was analysed by an immunofluorescence staining using an antibody against Ki67. AT2 cells of G4 *Tert* ^*−/−*^ lungs showed significantly reduced proliferation rate compared to AT2 cells of control mice on post‐PNX Day 5 (Figure 2D,E).

On post‐PNX Day 21, we investigate the AT2 cell differentiation by an immunofluorescence staining experiment using antibodies against the AT1 marker HOPX and GFP in control and G4 *Tert* ^*−/−*^ lungs. In control lungs, a large number of HOPX^+^GFP^+^ cells, which are derived from AT2 cells, showed long and flat AT1 cell phenotypes (Figure 2F). G4 *Tert* ^*−/−*^ lungs had fewer HOPX^+^GFP^+^ cells than control lungs, and many of the G4 *Tert* ^*−/−*^ HOPX^+^GFP^+^ cells were not elongated. We quantified both HOPX^+^GFP^+^ cells and GFP^+^ cells, and found that the percentage of AT2 cell‐derived AT1 cells in G4 *Tert* ^*−/−*^ mice was significantly reduced compared to control lungs on post‐PNX Day 21 (Figure 2G). Together, these results indicated that post‐PNX alveolar regeneration is impaired in G4 *Tert* ^*−/−*^ mice.

### 3.3 The impaired regeneration of G4 *Tert* ^*−/−*^ AT2 cells cannot be rescued by normal stromal cells

**FIGURE 3:**
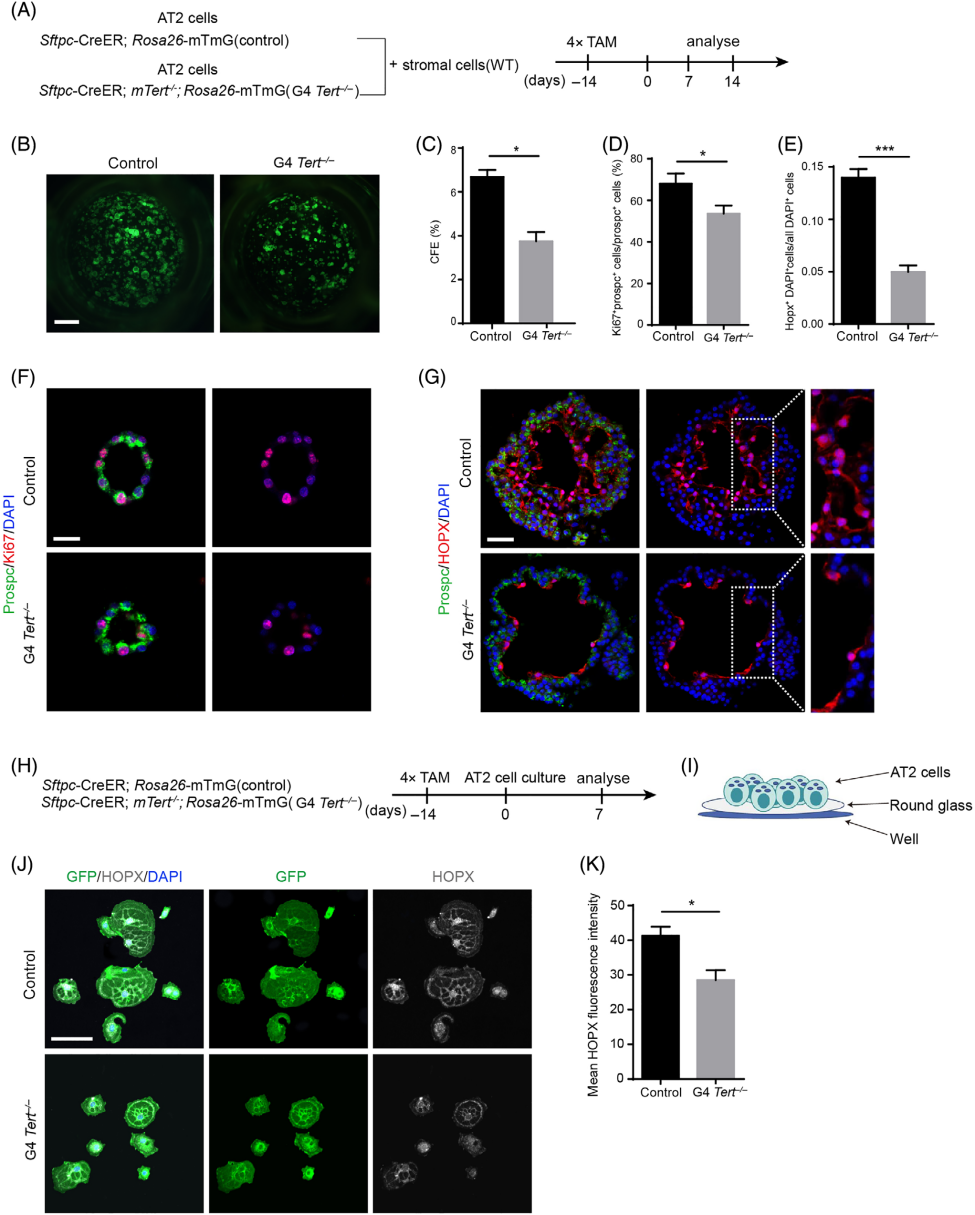
G4 *Tert* ^*−/−*^ AT2 cells show impaired proliferation and differentiation in vitro. (A) Analysis of the alveolar organoids culture of control and G4 *Tert* ^*−/−*^ AT2 cells at Days 7 and 14. (B) Image of alveolar organoids of control and G4 *Tert* ^*−/−*^ mice at Day 14. (C–E) Quantification of the colony forming efficiency (CFE) (*n* = 3) (C), the percentage of Ki67^+^prospc^+^ cells/all prospc^+^ cells (D) and HOPX^+^DAPI^+^ cells/all DAPI^+^ cells (E) (mean ± SEM, *n* = 6). (F) Alveolar organoids were stained with antibodies against prospc, Ki67 at Day 7. (G) Alveolar organoids were stained with antibodies against prospc, HOPX at Day 14. (H) Analysis of 2D cell culture of control and G4 *Tert* ^*−/−*^ AT2 cells at Day 7. (I) Diagram of the 2D cell culture model of AT2 cells in vitro. (J) Cells were stained with antibodies against HOPX and GFP at Day 7. (K) Quantification of mean HOPX fluorescence intensity of control and G4 *Tert* ^*−/−*^ cells (mean ± SEM, *n* = 10). *p < 0.05, **p < 0.01, ***p < 0.001, n.s., not significant. Student's *t*‐test. Scale bars represent 20 μm (F), 50 μm (G,J), and 1 mm (B)

Because of the loss of *Tert* in all cells in our mouse model, we could not exclude the effect of stromal cells with *Tert* deficiency on the regeneration of AT2 cells in G4 *Tert* ^*−/−*^ mice. We therefore set up an alveolar organoid system by culturing wild type stromal cells with G4 *Tert* ^*−/−*^ AT2 cells. We separately cultured AT2 cells of control or G4 *Tert* ^*−/−*^ mice with stromal cells that were collected from control mice, and analysed the alveolar spheres on Days 7 and 14 (Figure 3A). We found that AT2 cells in G4 *Tert* ^*−/−*^ mice generated fewer organoids when co‐cultured with wild type Pdgfrα^+^ stromal cells (Figure 3B). The colony forming efficiency (CFE) in G4 *Tert* ^*−/−*^ AT2 cells was significantly reduced (Figure 3C). By immunofluorescence staining experiments, we found that the proliferation and differentiation of AT2 cells in G4 *Tert* ^*−/−*^ mice were impaired when co‐cultured with wild type stromal cells (Figure 3D–G).

To further explore whether the impaired differentiation of G4 *Tert* ^*−/−*^ AT2 cells is independent of their reduced proliferation, we constructed a 2D cell culture model for AT2 cells. AT2 cells isolated by FACS were cultured on the glass in vitro (Figure 3I). In this culture model, AT2 cells can differentiate into AT1 cells without proliferation. We then evaluated the differentiation in G4 *Tert* ^*−/−*^ AT2 cells by immunofluorescence staining against GFP and HOPX on Day 7 (Figure 3H). Compared to the control AT2 cells, the expression level of HOPX in G4 *Tert* ^*−/−*^ group was significantly reduced (Figure 3J,K), indicating the impaired differentiation of G4 *Tert* ^*−/−*^ AT2 cells. These results demonstrated that G4 *Tert* ^*−/−*^ AT2 cells have limited self‐renewal and differentiation capacity and the impaired differentiation of G4 *Tert* ^*−/−*^ AT2 cells is independent of their decreased proliferation.

### 3.4 The gene expression that regulates cytoskeleton remodelling is decreased in G4 *Tert* ^*−/−*^ AT2 cells on post‐PNX Day 14

**FIGURE 4:**
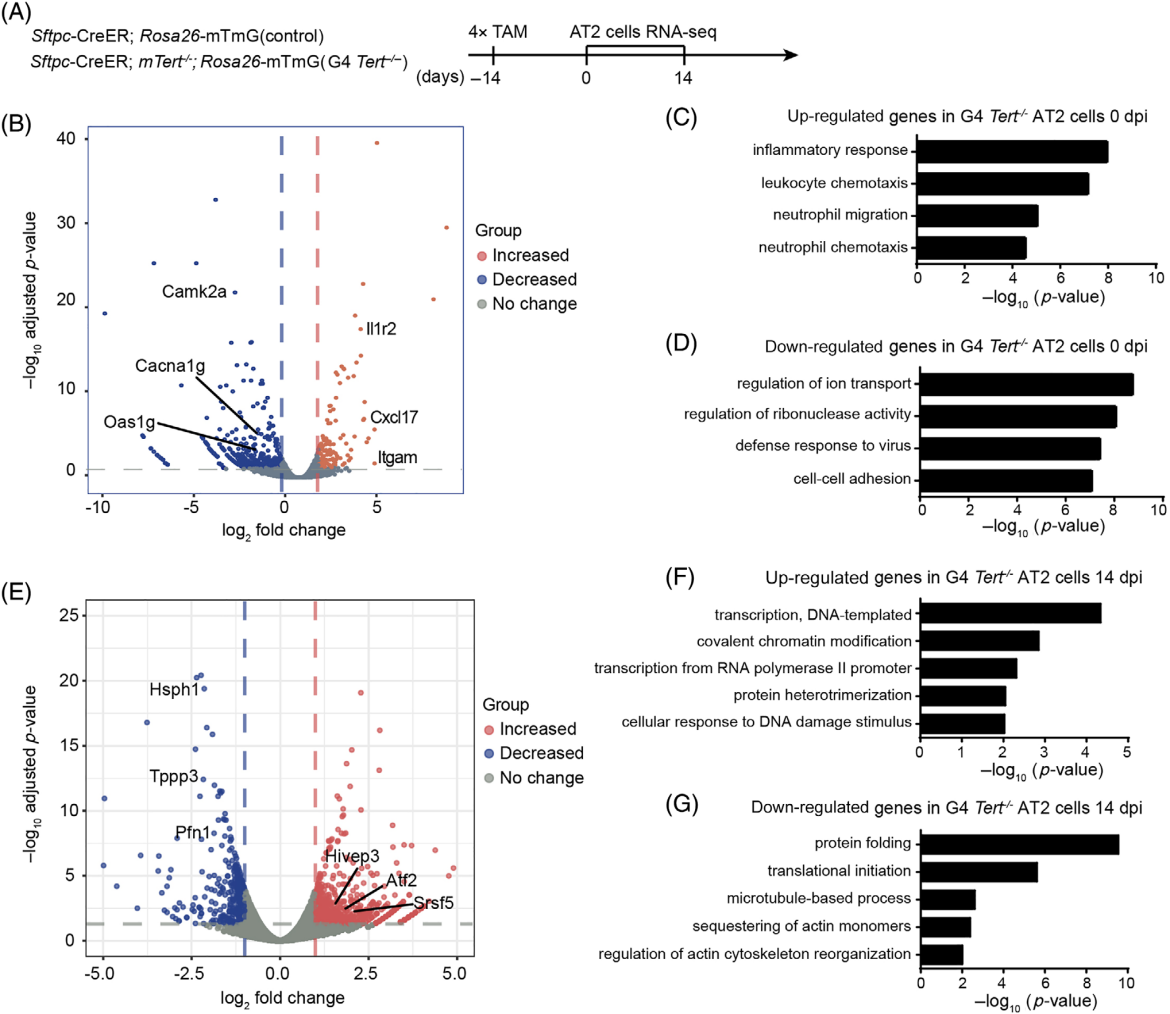
Genes that regulated cytoskeleton remodelling were down‐regulated in G4 *Tert* ^*−/−*^ AT2 cells. (A) Analysis of RNA sequencing of control and G4 *Tert* ^*−/−*^ AT2 cells on post‐PNX Days 0 and 14. (B,E) Volcano plot of the differentially expressed genes (DEGs) of control and G4 *Tert* ^*−/−*^ AT2 cells on post‐PNX Days 0 and 14. (C,D) Functional enrichment analysis with GO biological processes of up‐ and down‐regulated genes in G4 *Tert* ^*−/−*^ AT2 cells on post‐PNX Day 0. (F,G) Functional enrichment analysis with GO biological processes of up‐ and down‐regulated genes in G4 *Tert* ^*−/−*^ AT2 cells on post‐PNX Day 14. The *X*‐axis represents the −log_10_ (*p*‐value)

To further understand the mechanisms underlying G4 *Tert* ^*−/−*^ AT2 cell‐mediated defective regeneration, we performed RNA‐seq analysis to characterize the DEGs in G4 *Tert* ^*−/−*^ AT2 cells compared to control mice. According to a previous study,^32^ the differentiation of AT2 cells was observed on post‐PNX Day 14. Therefore, we analysed the DEGs in the differentiation of G4 *Tert* ^*−/−*^ AT2 cells on post‐PNX Day 14 (Figure 4A). Among a total of 1742 DEGs on post‐PNX Day 14, 514 genes were up‐regulated and 1228 were down‐regulated in G4 *Tert* ^*−/−*^ AT2 cells (Figure 4E). GO analysis showed that the up‐regulated genes exhibited enrichment for genes with functional annotations related to the following terms: regulation of transcription, covalent chromatin modification, protein heterotrimerization and cellular response to DNA damage stimulus (Figure 4F). The down‐regulated DEGs showed enrichment for genes involved in protein folding, protein synthesis, cytoskeleton remodelling like microtubule‐based process, sequestering of actin monomers and regulation of actin cytoskeleton reorganization (Figure 4G). Microtubule and actin filaments form the cytoskeleton of cells together with microfilaments in the cytoplasm of eukaryotic cells.^34^, ^35^ The cytoskeleton is a complex, dynamic network of proteins, which not only functions directly in maintaining cell morphology, but also participates in many important cell activities. We hypothesized that cytoskeleton remodelling may be impaired during the differentiation of AT2 cells in G4 *Tert* ^*−/−*^ mice. We also analysed the DEGs of G4 *Tert* ^*−/−*^ AT2 cells compared to control AT2 cells without PNX (Figure 4B). We found that the gene expression of inflammatory response was up‐regulated, while the regulation of ion transport and regulation of ribonuclease activity were down‐regulated in G4 *Tert* ^*−/−*^ AT2 cells (Figure 4C,D).

### 3.5 The cytoskeleton remodelling is defective in the differentiation of G4 *Tert* ^*−/−*^ AT2 cells

**FIGURE 5:**
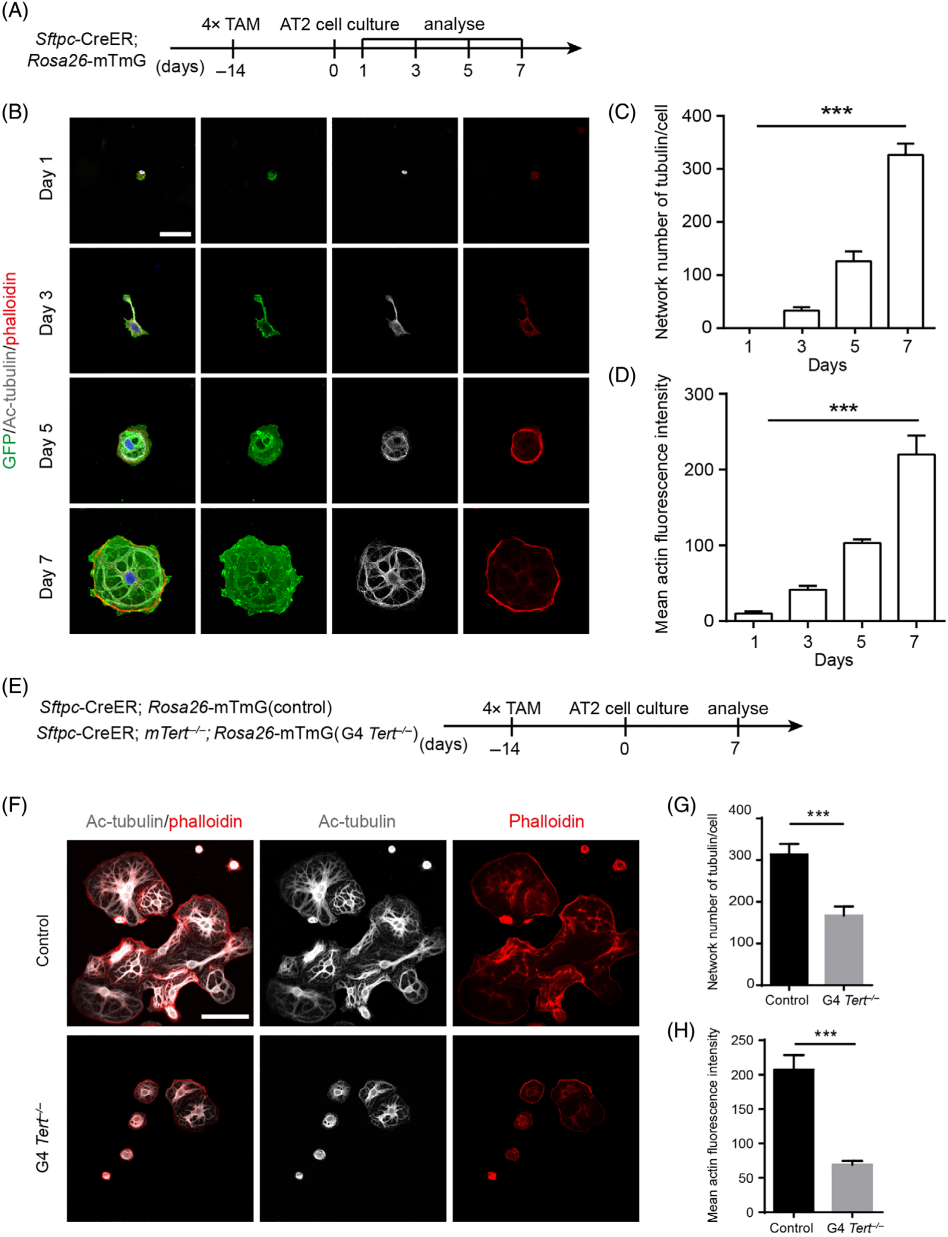
Defective cytoskeleton remodelling during the differentiation of G4 *Tert* ^*−/−*^ AT2 cells. (A) Analysis of acetylated‐tubulin (Ac‐tubulin) and actin of control AT2 cells at Days 1, 3, 5 and 7 in 2D cell culture model. (B) Cells were stained with antibodies against phalloidin, Ac‐tubulin and GFP at Days 1, 3, 5 and 7. (C,D) Quantification of the network number of microtubules in each cell and the mean actin fluorescence intensity of control AT2 cells at Days 1, 3, 5 and 7 (mean ± SEM, *n* = 10). (E) Analysis of the differentiation of control and G4 *Tert* ^*−/−*^ AT2 cells at Day 7. (F) Cells were stained with antibodies against phalloidin and Ac‐tubulin of control and G4 *Tert* ^*−/−*^ AT2 cells at Day 7. (G,H) Quantification of the network number of microtubules in each cell and the mean actin fluorescence intensity of control and G4 *Tert* ^*−/−*^ mice at Day 7 (mean ± SEM, *n* = 10). *p < 0.05, **p < 0.01, ***p < 0.001, n.s., not significant. Student's *t*‐test. Scale bars represent 20 μm (B), 50 μm (F)

To investigate the cytoskeleton remodelling of AT2 cells in G4 *Tert* ^*−/−*^ mice, we cultured AT2 cells isolated from control and G4 *Tert* ^*−/−*^ mice in a 2D culture system, and evaluated cytoskeleton remodelling by immunofluorescence staining against acetylated‐tubulin (Ac‐tubulin) and phalloidin (Figure 5A).

We found that the cytoskeleton was rapidly remodelled followed by the shape changes of AT2 cells during control AT2 cell differentiation. As shown in Figure 5B, the networks of microtubule and actin contributed to changes in cell shape during the differentiation of AT2 cells. AT2 cells became irregular shaped and showed small cellular projections, and ultimately formed a complex and orderly cytoskeleton structure. The network number of microtubules and the expression level of actin were gradually increased during the differentiation of AT2 cells (Figure 5C,D). On Day 7, the cytoskeleton structure of flattened AT1 cells is established.

We then analysed the cytoskeleton remodelling in G4 *Tert* ^*−/−*^ AT2 cells on Day 7 (Figure 5E). Compared to the well‐organized and stable microtubule structure in control AT2 cells, the microtubule structure of G4 *Tert* ^*−/−*^ cells was disorganized, and the network number of microtubules in cells was decreased (Figure 5F,G). The expression levels of polymerized actin in G4 *Tert* ^*−/−*^ cells were also significantly reduced (Figure 5H). Therefore, we demonstrated that the cytoskeleton remodelling is impaired during the differentiation of G4 *Tert* ^*−/−*^ AT2 cells.

### 3.6 Elevated TPPP3 expression follows the microtubule remodelling during the differentiation of AT2 cells

**FIGURE 6:**
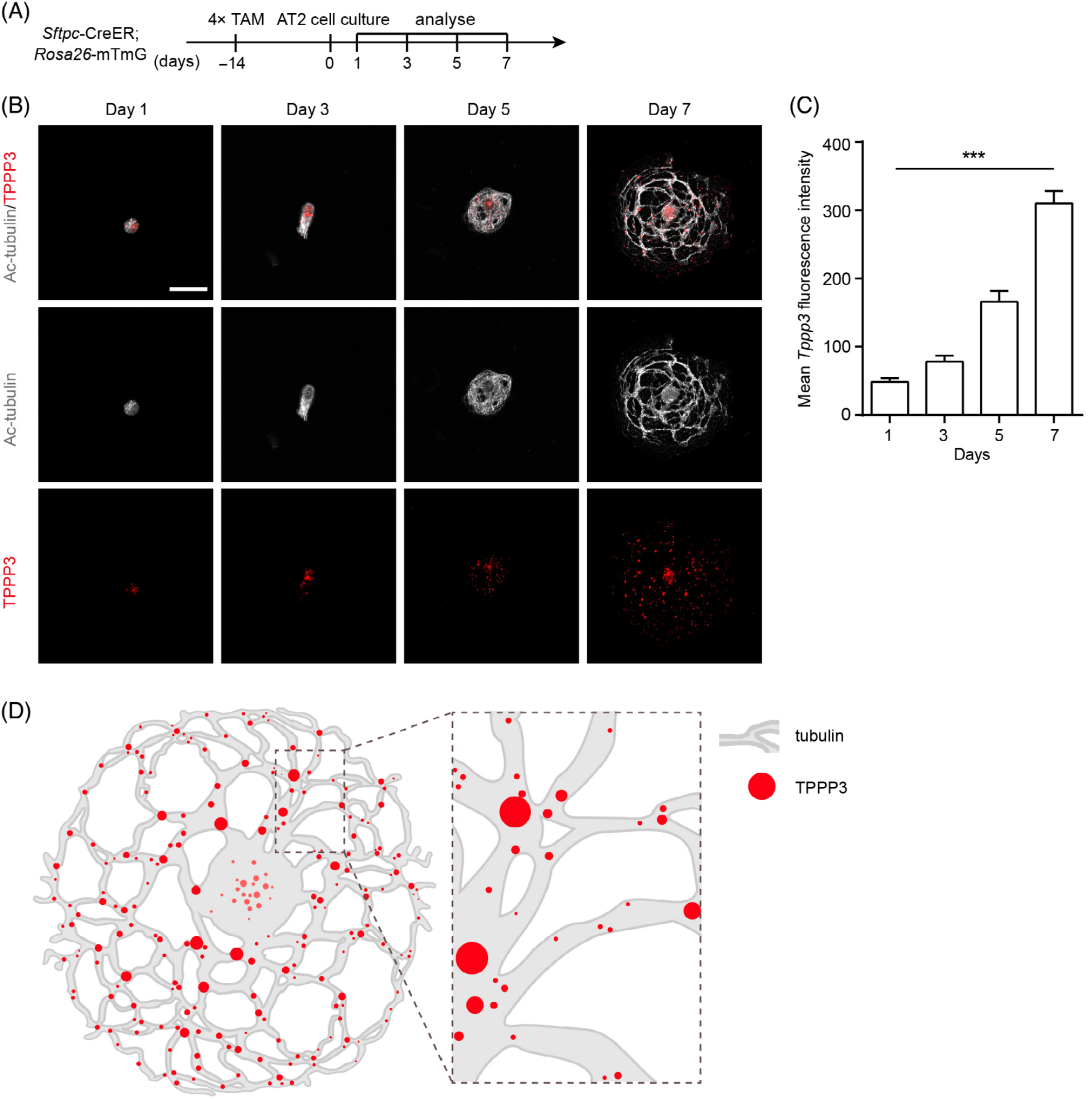
The expression of TPPP3 is gradually increased during the differentiation of AT2 cells. (A) Analysis of Ac‐tubulin and TPPP3 of control AT2 cells at Days 1, 3, 5 and 7 in the 2D cell culture model. (B) Cells were stained with antibodies against Ac‐tubulin and TPPP3 at Days 1, 3, 5 and 7. (C) Quantification of the mean TPPP3 fluorescence intensity of control AT2 cells at Days 1, 3, 5 and 7 (mean ± SEM, *n* = 10). (D) The schematic diagram of TPPP3 located on the microtubules of control AT2 cells at Day 7 in the 2D cell culture model

According to our RNA‐seq results, the gene expression of *Tppp3* was decreased during the differentiation of G4 *Tert* ^*−/−*^ AT2 cells. Previous studies showed that the loss of *Tppp3* results in defective cytoskeleton remodelling.^28^ Thus we next want to further investigate the expression of TPPP3 during the differentiation of AT2 cells. We cultured control AT2 cells in the 2D culture system and characterized the expression levels of TPPP3 by immunofluorescence staining experiments using antibodies against Ac‐tubulin and TPPP3 at Days 1, 3, 5 and 7 (Figure 6A). We found that TPPP3 is initially located on the microtubule near the nucleus, and then the expression level of TPPP3 was gradually increased, following the microtubule remodelling during the differentiation of AT2 cells. The TPPP3 eventually distributes broadly on the microtubule networks at Day 7 (Figure 6B,C). As shown in Figure 6D, TPPP3 mostly located on the trunk or intersections of microtubules on Day 7, suggesting the role of TPPP3 in maintaining the integrity and stability of microtubules. We then hypothesized that TPPP3 may be required for the microtubule remodelling and the differentiation of AT2 cells.

### 3.7 TPPP3‐ mediated microtubule remodelling is essential for AT2 cell differentiation

**FIGURE 7:**
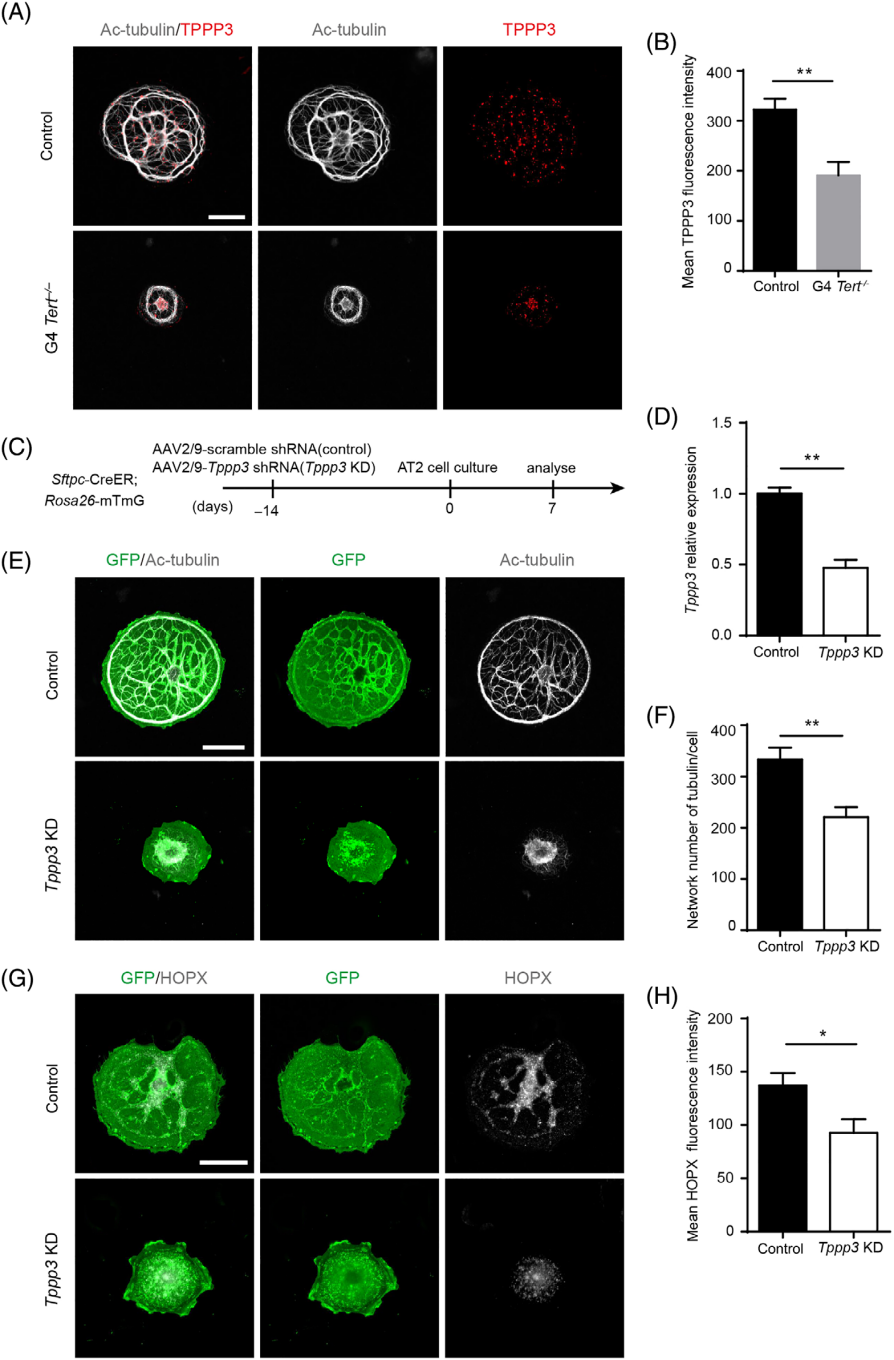
TPPP3‐mediated microtubule remodelling is required for AT2 cell differentiation. (A) Cells of control and G4 *Tert* ^*−/−*^ AT2 cells were stained with antibodies against Ac‐tubulin and TPPP3 at Day 7. (B) Quantification of the mean TPPP3 fluorescence intensity of control and G4 *Tert* ^*−/−*^ cells (mean ± SEM, *n* = 10). (C) AAV2/9 viruses expressing either *Tppp3* shRNA (*Tppp3* KD) or scramble shRNA (control) were delivered into the mouse lungs before collected AT2 cells for 2D cell culture. (D) The relative gene expression of *Tppp3* in AT2 cells collected from mouse lungs treated with either of the viruses. (E) Cells were stained with antibodies against Ac‐tubulin and GFP of control and *Tppp3* KD group at Day 7. (F,H) Quantification of the network number of microtubules in each cell and mean HOPX fluorescence intensity of control and *Tppp3* KD mice at Day 7 (mean ± SEM, *n* = 10). (G) Cells were stained with antibodies against HOPX and GFP of control and *Tppp3* KD group at Day 7. *p < 0.05, **p < 0.01, ***p < 0.001, n.s., not significant. Student's *t*‐test. Scale bars represent 20 μm (A,E,G)

We further compared the expression levels of TPPP3 in the control and G4 *Tert* ^*−/−*^ AT2 cells by immunofluorescence staining experiments. Our results showed that the fluorescence intensity of TPPP3 in G4 *Tert* ^*−/−*^ cells was significantly lower than that of the control cells (Figure 7A,B). We then investigated the effects of reducing TPPP3 on the differentiation of AT2 cells. We delivered AAV2/9 viruses expressing either *Tppp3* short hairpin RNA (shRNA) (*Tppp3* KD) or scramble shRNA (control) into the lungs of *Sftpc*‐CreER; *Rosa26*‐mTmG mice intratracheally. AT2 cells of treated mice were isolated and cultured on the glass until Day 7 in vitro (Figure 7C). Compared to the control AT2 cells, the expression of *Tppp3* was significantly reduced in *Tppp3* KD AT2 cells (Figure 7D). We found that the microtubule structure in *Tppp3* KD cells was disorganized, and the number of microtubule networks in these cells was significantly decreased compared to control mice (Figure 7E,F). Furthermore, the expression level of HOPX was reduced in *Tppp3* KD cells (Figure 7G,H), indicating the impaired differentiation of *Tppp3* KD AT2 cells. Together, these results reveal that TPPP3 is essential for the microtubule remodelling during AT2 cells differentiation.

## 4 DISCUSSION

In this study, we generate G4 *Tert* ^*−/−*^ mice, in which the telomere length is shorter compared to control mice. The proliferation and differentiation of AT2 cells of G4 *Tert* ^*−/−*^ mice are significantly decreased, and co‐culturing G4 *Tert* ^*−/−*^ AT2 cells with wild type stromal cells cannot rescue the defects of G4 *Tert* ^*−/−*^ AT2 cells. RNA‐seq results revealed that the expression levels of many genes that regulate the cytoskeleton remodelling are decreased in G4 *Tert* ^*−/−*^ AT2 cells. We demonstrated that the cytoskeleton remodelling of G4 *Tert* ^*−/−*^ AT2 cells was defective.

The pathogenesis of lung diseases has been related to AT2 cells with short telomeres.^36^, ^37^, ^38^ Short telomere caused by telomere related gene mutations is closely related to the pathogenesis of interstitial lung disease,^37^, ^39^ of which the most common one is IPF. Armanios found that *TERT* and *TERC* mutations are present in 8% of patients with familial IPF precursors, which have been found in patients with sporadic IPF. Other studies have found that up to 30% of patients with familial IPF have shortened telomere length and/or carry telomere related gene mutations.^40^ In the larger cohort of sporadic IPF, about 10% of patients have telomere associated mutations, the most common being *TERT*.^41^ Previous studies suggested that AT2 cells with short telomeres are relevant to the increased susceptibility to fibrosis and emphysema in mice.^8^, ^9^ Gao et al. further confirmed that increased expression of telomeric repeat‐containing RNA (TERRA) in AT2 cells of bleomycin‐induced pulmonary fibrosis mice can lead to increased oxidative stress and apoptosis, and TERRA inactivation can ameliorate bleomycin‐induced pulmonary fibrosis in mice.^42^ Alder et al. found that short telomeres in AT2 cells of *Terc* ^*−/−*^ mice limited alveolar organoids formation.^43^ A recent study showed that overexpression of telomere protection protein 1 (TPP1) lengthened telomeres, expanded the AT2 cells population and inhibited bleomycin‐induced pulmonary fibrosis and respiratory dysfunction.^44^

The most common manifestation of short telomere related‐lung diseases is IPF. The impaired alveolar regeneration is recognized as the important pathogenesis of IPF.^31^ Few studies elaborated the relationships between short telomeres and the regeneration of AT2 cells. Liu and Lee showed that telomere shortening led to AT2 cells senescence and apoptosis, and there are fewer AT2 cells in the lung of short telomere mice in homeostasis, suggesting that fewer AT2 cells can be triggered to regenerate after injury.^45^, ^46^ Our studies also found that the ability of regeneration of AT2 cells is defective by genetic lineage tracing. Together, these two points can account for the impaired alveolar regeneration of short telomere mice. Armanios and co‐authors reported that short telomeres in AT2 cells limited alveolosphere formation, which are susceptibility to injury that are characteristic of telomere‐mediated lung disease.^43^ However, the underlying mechanisms of impaired regeneration are poorly understood.

Our studies reveal that short telomere can lead to the impaired alveolar regeneration, closely related to the immature cytoskeleton remodelling. We show that the cytoskeleton networks remodel significantly during the differentiation of AT2 cells. Our RNA‐seq analysis showed decreased expression levels of genes that regulated cytoskeleton remodelling in G4 *Tert* ^*−/−*^ AT2 cells on post‐PNX Day 14. Interestingly, the expression level of TPPP3 was decreased in G4 *Tert* ^*−/−*^ AT2 cells in vitro.

The cytoskeleton is a protein filament network formed by microtubules, actin and intermediate filaments.^47^, ^48^, ^49^ It functions as establish and stabilize cell shape, maintains structural integrity, and participates in cell division, migration and other biological functions.^50^, ^51^, ^52^ In the cell fate, cytoskeleton plays a vital role in the cell growth and development. It is well known that neural development is particularly reliant on functional microtubule structures. TPPP3 is a member of the tubulin polymerization promoting protein family.^53^, ^54^ It is involved in microtubule binding and stabilization of existing microtubules, thus maintaining the integrity of the microtubule network.^26^, ^27^ Previous studies found that *Tppp* null oligodendrocytes have defects in microtubule organization.^28^ It is also indicated that TPPPs are required for axon regeneration in central and periphery nervous system after injury,^29^ but few studies revealed the role of cytoskeleton in alveolar regeneration.

Thus, we demonstrate that TPPP3 is critical for both the microtubule remodelling and the differentiation of AT2 cells. We propose that the short telomeres induced by *Tert* deletion in AT2 cells reduce the expression of TPPP3, eventually leading to the defective microtubule remodelling and the impaired differentiation of AT2 cells. It remains a question whether TERT can directly regulate the expression of TPPP3 in AT2 cells. Further studies are still required to explore the regulatory mechanism of expression of TPPP3 in AT2 cells.

## CONFLICT OF INTEREST

The authors declare that they have no conflict of interest.

## AUTHOR CONTRIBUTIONS

Xin Zhang performed the experiments, collected and assembled the data and wrote the manuscript. Mengting Shi and Xi Zhao performed the left lung pneumonectomy. Ennan Bin performed analysis of RNA‐seq data. Yucheng Hu helped to perform the data analysis. Nan Tang planned the project, analysed and interpreted the data and revised the manuscript. Huaping Dai and Chen Wang conceived and supervised the project, and interpreted the data and revised the manuscript. All authors contributed to the article and approved the submitted version.

## Data Availability

The data that support the findings of this study are available from the corresponding author upon reasonable request.

## References

[1] Chakravarti D , LaBella KA , DePinho RA . Telomeres: history, health, and hallmarks of aging. Cell. 2021;184(2):306‐322. doi:10.1016/j.cell.2020.12.028 33450206 PMC8081271

[2] Turner KJ , Vasu V , Griffin DK . Telomere biology and human phenotype. Cell. 2019;8(1):73. doi:10.3390/cells8010073 PMC6356320 30669451

[3] Roake CM , Artandi SE . Regulation of human telomerase in homeostasis and disease. Nat Rev Mol Cell Biol. 2020;21(7):384‐397. doi:10.1038/s41580-020-0234-z 32242127 PMC7377944

[4] Herrmann M , Pusceddu I , Marz W , Herrmann W . Telomere biology and age‐related diseases. Clin Chem Lab Med. 2018;56(8):1210‐1222. doi:10.1515/cclm-2017-0870 29494336

[5] Sanchez‐Vazquez R , Guío‐Carrión A , Zapatero‐Gaviria A , Martínez P , Blasco MA . Shorter telomere lengths in patients with severe COVID‐19 disease. Aging. 2021;13(1):1‐15. doi:10.18632/aging.202463 33428591 PMC7835063

[6] Stock SCJW , Renzoni EA . Telomeres in interstitial lung disease. J Clin Med. 2021;10(7). doi:10.3390/jcm10071384 PMC8037770 33808277

[7] Snetselaar R , van Batenburg AA , van Oosterhout MFM , et al. Short telomere length in IPF lung associates with fibrotic lesions and predicts survival. PLoS One. 2017;12(12):e0189467. doi:10.1371/journal.pone.0189467.t001 29281671 PMC5744955

[8] Alder JK , Guo N , Kembou F , et al. Telomere length is a determinant of emphysema susceptibility. Am J Respir Crit Care Med. 2011;184(8):904‐912. doi:10.1164/rccm.201103-0520OC 21757622 PMC3208661

[9] Liu YY , Shi Y , Liu Y , Pan XH , Zhang KX . Telomere shortening activates TGF‐beta/Smads signaling in lungs and enhances both lipopolysaccharide and bleomycin‐induced pulmonary fibrosis. Acta Pharmacol Sin. 2018;39(11):1735‐1745. doi:10.1038/s41401-018-0007-9 29925920 PMC6289325

[10] Povedano JM , Martinez P , Serrano R , et al. Therapeutic effects of telomerase in mice with pulmonary fibrosis induced by damage to the lungs and short telomeres. elife. 2018;7:e31299. doi:10.7554/eLife.31299.001 29378675 PMC5818250

[11] Beers MF , Moodley Y . When is an alveolar type 2 cell an alveolar type 2 cell? A conundrum for lung stem cell biology and regenerative medicine. Am J Respir Cell Mol Biol. 2017;57(1):18‐27. doi:10.1165/rcmb.2016-0426PS 28326803 PMC5516281

[12] Wu H , Tang N . Stem cells in pulmonary alveolar regeneration. Development. 2021;148(2):dev193458. doi:10.1242/dev.193458 33461972

[13] Gupta SK , Srivastava M , Minocha R , Akash A , Dangwal S , Dandekar T . Alveolar regeneration in COVID‐19 patients: a network perspective. Int J Mol Sci. 2021;22(20):11279. doi:10.3390/ijms222011279 PMC8538208 34681944

[14] Jones‐Freeman B , Starkey MR . Bronchioalveolar stem cells in lung repair, regeneration and disease. J Pathol. 2020;252(3):219‐226. doi:10.1002/path.5527 32737996

[15] Chen J , Wu H , Yu Y , Tang N . Pulmonary alveolar regeneration in adult COVID‐19 patients. Cell Res. 2020;30(8):708‐710. doi:10.1038/s41422-020-0369-7 32632255 PMC7338112

[16] Katzen J , Beers MF . Contributions of alveolar epithelial cell quality control to pulmonary fibrosis. J Clin Investig. 2020;130(10):5088‐5099. doi:10.1172/jci139519 32870817 PMC7524463

[17] Parimon T , Yao C , Stripp BR , Noble PW , Chen P . Alveolar epithelial type II cells as drivers of lung fibrosis in idiopathic pulmonary fibrosis. Int J Mol Sci. 2020;21(7):2269. doi:10.3390/ijms21072269 PMC7177323 32218238

[18] Janke C , Bulinski JC . Post‐translational regulation of the microtubule cytoskeleton: mechanisms and functions. Nat Rev Mol Cell Biol. 2011;12(12):773‐786. doi:10.1038/nrm3227 22086369

[19] Pegoraro AF , Janmey P , Weitz DA . Mechanical properties of the cytoskeleton and cells. Cold Spring Harb Perspect Biol. 2017;9(11):a022038. doi:10.1101/cshperspect.a022038 PMC5666633 29092896

[20] Pollard TD , Goldman RD . Overview of the cytoskeleton from an evolutionary perspective. Cold Spring Harb Perspect Biol. 2018;10(7):a030288. doi:10.1101/cshperspect.a030288 PMC6028065 29967009

[21] Wu J , Akhmanova A . Microtubule‐organizing centers. Annu Rev Cell Dev Biol. 2017;33:51‐75. doi:10.1146/annurev-cellbio-100616-060615 28645217

[22] Bodakuntla S , Jijumon AS , Villablanca C , Gonzalez‐Billault C , Janke C . Microtubule‐associated proteins: structuring the cytoskeleton. Trends Cell Biol. 2019;29(10):804‐819. doi:10.1016/j.tcb.2019.07.004 31416684

[23] Dogterom M , Koenderink GH . Actin‐microtubule crosstalk in cell biology. Nat Rev Mol Cell Biol. 2019;20(1):38‐54. doi:10.1038/s41580-018-0067-1 30323238

[24] Théry M , Blanchoin L . Microtubule self‐repair. Curr Opin Cell Biol. 2021;68:144‐154. doi:10.1016/j.ceb.2020.10.012 33217636

[25] Olah J , Lehotzky A , Szunyogh S , Szenasi T , Orosz F , Ovadi J . Microtubule‐associated proteins with regulatory functions by day and pathological potency at night. Cell. 2020;9(2):357. doi:10.3390/cells9020357 PMC7072251 32033023

[26] Vincze O , Tökési N , Oláh J , et al. Tubulin polymerization promoting proteins (TPPPs): members of a new family with distinct structures and functions. Biochemistry. 2006;45(46):13818‐13826. doi:10.1021/bi061305e 17105200

[27] Olah J , Szenasi T , Szabo A , et al. Tubulin binding and polymerization promoting properties of tubulin polymerization promoting proteins are evolutionarily conserved. Biochemistry. 2017;56(7):1017‐1024. doi:10.1021/acs.biochem.6b00902 28106390

[28] Fu MM , McAlear TS , Nguyen H , et al. The golgi outpost protein TPPP nucleates microtubules and is critical for myelination. Cell. 2019;179(1):132‐146 e14. doi:10.1016/j.cell.2019.08.025 31522887 PMC7214773

[29] Vargas EJM , Matamoros AJ , Qiu J , et al. The microtubule regulator ringer functions downstream from the RNA repair/splicing pathway to promote axon regeneration. Genes Dev. 2020;34(3–4):194‐208. doi:10.1101/gad.331330.119 31919191 PMC7000917

[30] Chen R , Zhang K , Chen H , et al. Telomerase deficiency causes alveolar stem cell senescence‐associated low‐grade inflammation in lungs. J Biol Chem. 2015;290(52):30813‐30829. doi:10.1074/jbc.M115.681619 26518879 PMC4692211

[31] Wu H , Yu Y , Huang H , et al. Progressive pulmonary fibrosis is caused by elevated mechanical tension on alveolar stem cells. Cell. 2020;180(1):107‐121 e17. doi:10.1016/j.cell.2019.11.027 31866069

[32] Liu Z , Wu H , Jiang K , et al. MAPK‐mediated YAP activation controls mechanical‐tension‐induced pulmonary alveolar regeneration. Cell Rep. 2016;16(7):1810‐1819. doi:10.1016/j.celrep.2016.07.020 27498861

[33] Erdmann N , Liu Y , Harrington L . Distinct dosage requirements for the maintenance of long and short telomeres in mTert heterozygous mice. Proc Natl Acad Sci U S A. 2004;101(16):6080‐6085. doi:10.1073/pnas.0401580101 15079066 PMC395926

[34] LaFlamme SE , Mathew‐Steiner S , Singh N , Colello‐Borges D , Nieves B . Integrin and microtubule crosstalk in the regulation of cellular processes. Cell Mol Life Sci. 2018;75(22):4177‐4185. doi:10.1007/s00018-018-2913-x 30206641 PMC6182340

[35] Roll‐Mecak A . The tubulin code in microtubule dynamics and information encoding. Dev Cell. 2020;54(1):7‐20. doi:10.1016/j.devcel.2020.06.008 32634400 PMC11042690

[36] Cagsin H , Uzan A , Tosun O , Rasmussen F , Serakinci N . Tissue‐specific ultra‐short telomeres in chronic obstructive pulmonary disease. Int J Chron Obstruct Pulmon Dis. 2020;15:2751‐2757. doi:10.2147/COPD.S267799 33154635 PMC7608580

[37] Courtwright AM , El‐Chemaly S . Telomeres in interstitial lung disease: the short and the long of it. Ann Am Thorac Soc. 2019;16(2):175‐181. doi:10.1513/AnnalsATS.201808-508CME 30540921 PMC6376948

[38] Duckworth A , Gibbons MA , Allen RJ , et al. Telomere length and risk of idiopathic pulmonary fibrosis and chronic obstructive pulmonary disease: a Mendelian randomisation study. Lancet Respir Med. 2021;9(3):285‐294. doi:10.1016/S2213-2600(20)30364-7 33197388

[39] Arish N , Petukhov D , Wallach‐Dayan SB . The role of telomerase and telomeres in interstitial lung diseases: from molecules to clinical implications. Int J Mol Sci. 2019;20(12):2996. doi:10.3390/ijms20122996 PMC6627617 31248154

[40] Uitto J , Bauer EA , Moshell AN . Symposium on epidermolysis bullosa: molecular biology and pathology of the cutaneous basement membrane zone. Jefferson Medical College, Philadelphia, Pennsylvania, October 4 and 5, 1991. J Invest Dermatol. 1992;98(3):391‐395. doi:10.1111/1523-1747.ep12499822 1545149

[41] Hoffman TW , van Moorsel CHM , Borie R , Crestani B . Pulmonary phenotypes associated with genetic variation in telomere‐related genes. Curr Opin Pulm Med. 2018;24(3):269‐280. doi:10.1097/mcp.0000000000000475 29474209

[42] Gao Y , Zhang J , Liu Y , et al. Regulation of TERRA on telomeric and mitochondrial functions in IPF pathogenesis. BMC Pulm Med. 2017;17(1):163. doi:10.1186/s12890-017-0516-1 29197377 PMC5712138

[43] Alder JK , Barkauskas CE , Limjunyawong N , et al. Telomere dysfunction causes alveolar stem cell failure. Proc Natl Acad Sci U S A. 2015;112(16):5099‐5104. doi:10.1073/pnas.1504780112 25840590 PMC4413294

[44] Wang L , Chen R , Li G , et al. FBW7 mediates senescence and pulmonary fibrosis through telomere uncapping. Cell Metab. 2020;32(5):860‐877.e9. doi:10.1016/j.cmet.2020.10.004 33086033

[45] Zhang K , Wang L , Chen H , et al. Pulmonary alveolar stem cells undergo senescence, apoptosis and differentiation by p53‐dependent and ‐independent mechanisms in telomerase deficient mice. Clin Exp Pharmacol Physiol. 2021;48(5):651‐659. doi:10.1111/1440-1681.13472 33634502

[46] Lee J , Reddy R , Barsky L , et al. Lung alveolar integrity is compromised by telomere shortening in telomerase‐null mice. Am J Physiol Lung Cell Mol Physiol. 2009;296(1):L57‐L70. doi:10.1152/ajplung.90411.2008 18952756 PMC2636955

[47] Goodson HV , Jonasson EM . Microtubules and microtubule‐associated proteins. Cold Spring Harb Perspect Biol. 2018;10(6):a022608. doi:10.1101/cshperspect.a022608 PMC5983186 29858272

[48] Svitkina T . The actin cytoskeleton and actin‐based motility. Cold Spring Harb Perspect Biol. 2018;10(1):a018267. doi:10.1101/cshperspect.a018267 PMC5749151 29295889

[49] Goldmann WH . Intermediate filaments and cellular mechanics. Cell Biol Int. 2018;42(2):132‐138. doi:10.1002/cbin.10879 28980752

[50] Janke C , Magiera MM . The tubulin code and its role in controlling microtubule properties and functions. Nat Rev Mol Cell Biol. 2020;21(6):307‐326. doi:10.1038/s41580-020-0214-3 32107477

[51] Kühn S , Mannherz HG . Actin: structure, function, dynamics, and interactions with bacterial toxins. Curr Top Microbiol Immunol. 2017;399:1‐34. doi:10.1007/82_2016_45 27848038

[52] Ohi R , Strothman C , Zanic M . Impact of the ‘tubulin economy’ on the formation and function of the microtubule cytoskeleton. Curr Opin Cell Biol. 2021;68:81‐89. doi:10.1016/j.ceb.2020.09.005 33160109 PMC7925340

[53] Staverosky JA , Pryce BA , Watson SS , Schweitzer R . Tubulin polymerization‐promoting protein family member 3, Tppp3, is a specific marker of the differentiating tendon sheath and synovial joints. Dev Dyn. 2009;238(3):685‐692. doi:10.1002/dvdy.21865 19235716

[54] Ye K , Li Y , Zhao W , et al. Knockdown of tubulin polymerization promoting protein family member 3 inhibits cell proliferation and invasion in human colorectal cancer. J Cancer. 2017;8(10):1750‐1758. doi:10.7150/jca.18943 28819371 PMC5556637

